# Supplementary material for: The Use of an Integrated Digital Tool to Improve the Efficiency of Multidisciplinary Tumor Boards—A Prospective Trial in Taiwan
Source: Cancers (Basel). 2025 Jan 28;17(3):444. doi: 10.3390/cancers17030444 (PMC11815928; doi:10.3390/cancers17030444)
Supplement: Supplementary file 1 [file cancers-17-00444-s001.zip › cancers-3392192-supplementary.pdf]

**Supplementary Table S1.** Steps performed by physicians and nurse case managers during the MDT process.

| Physicians | Nurse case managers | |
|---|---|---|
| | V | A-1 Establish case profile with basic data |
| | V | A-2 Collect all images for cancer staging |
| | V | A-3 Check all images to complete cancer staging |
| | V | A-4 Email meeting materials to MDT members |
| | V | Inform and schedule necessary diagnostic study or pathology review |
| | V | B-1 Obtained imaging access authority of EMR |
| V | | B-2 Present history |
| V | | B-3 Review key images for discussion |
| V | | B-4 Review pathology and additional IHC results |
| V | V | B-5 Comments from all related experts |
| V | | B-6 MDT conclusion |
| V | V | C-1 Complete EMR with MDT comments |
| V | V | D-1 Second follow-up discussion schedule |

EMR, electronic medical record; IHC, immunohistochemistry; MDT, multidisciplinary tumor board
